# Improvement in the ideal range of vault after implantable collamer lens implantation: a new vault prediction formula

**DOI:** 10.3389/fmed.2023.1132102

**Published:** 2023-04-27

**Authors:** Hao Wu, Ding-juan Zhong, Dong-qiang Luo, Li-yuan Zhang, Jia Liu, Hua Wang

**Affiliations:** The First Affiliated Hospital of Hunan Normal University/ Hunan Provincial People’s Hospital, Changsha, China

**Keywords:** implantable collamer lens, vault, ciliary sulcus morphology, AS-OCT, UBM, vault prediction formula

## Abstract

**Background:**

To derive and validate a novel vault prediction formula to improve the predictability and safety of implantable collamer lens (ICL) implantation.

**Methods:**

Thirty-five patients (61 eyes) with previous posterior chamber intraocular lens implantation were included. Various parameters, such as horizontal-visible iris diameter (HVID), photopic pupil diameter (PPD), axial length (AL), white-to-white (WTW), anterior chamber width (ACW), angle-to-angle (ATA), crystalline lens rise (CLR), anterior chamber depth (ACD), horizontal sulcus-to-sulcus (HSTS), and ciliary sulcus angle (CSA) were measured. Vault was measured at 3 months after surgery using CASIA2 anterior segment optical coherence tomography. The formula was derived using multiple linear regression analysis and named as WH formula. It was validated in 65 patients (118 eyes) to determine the percentage of the ideal postoperative vault range and to compare the differences between the WH formula and the NK, KS, and STAAR formulas.

**Results:**

Final ICL size, ATA, CSA, and CLR were included in the prediction formula model (adjusted *R*^2^ = 0.67, *p* < 0.001). The achieved vault 1 month after the surgery was 556.19 μm ± 166.98 μm in the validation group, and the ideal vault range was 200–800 μm (92%). The difference between the achieved vault and that predicted using the WH formula was not statistically significant (*p* = 0.165), whereas the difference between the achieved vault and that predicted using the NK and KS formulas was statistically significant (*p* < 0.001 and *p* < 0.001, respectively). The 95% agreement limit range of the achieved vault and the vault predicted using the WH formula was narrower than those predicted using the NK and KS formulas (−295.20–258.82 μm).

**Conclusion:**

This study combined the results of optical coherence tomography and ultrasound biomicroscopy measurements of the anterior segment of the eye and incorporated ciliary sulcus morphology quantification into the prediction formula. The study derived a prediction formula for vault by combining ICL size, ATA, and CLR. The derived formula was found to be superior to the currently available formulas.

## 1. Background

Implantable collamer lens (ICL) is an artificial lens implanted into the posterior chamber to correct a patient’s refractive error while preserving the lens itself. With the widespread use of ICL implantation, its safety has become a hot topic of clinical concern. The vault is the maximum vertical distance from the posterior surface of the ICL to the anterior surface of the lens, and whether ideal vault can be achieved after surgery is an important indicator of the safety of ICL surgery. When the vault is too high, the ICL tends to squeeze the peripheral anterior chamber, causing the closure of chamber angle and development of glaucoma (1). On the contrary, low vault can affect lens metabolism and lead to anterior subcapsular cataract (ASC) (2, 3). However, even after precise measurement of preoperative parameters, some of the operated eyes fail to have the correct vault after surgery.

It is now recognized that ICL size is the most important factor that affects postoperative vault (3–5). The ICL size is included in only four models (12.1 mm, 12.6 mm, 13.2 mm, and 13.7 mm), so it is necessary to optimize the preoperative lens size selection. Currently, most clinics use the formula provided by STAAR to select the lens size based on white-to-white (WTW) and anterior chamber depth (ACD) diameters. However, as the ICL is implanted in the posterior chamber, the complexity of the internal structures of the eye could not be taken into account based on WTW and ACD alone. The shape of the ciliary sulcus also affects the vault; thus, some patients do not achieve the desired vault after surgery (6, 7). Ultrasound biomicroscopy (UBM) is used clinically for sulcus-to-sulcus (STS) measurements and is the only method that can reveal posterior chamber morphology and ciliary body in the *in vivo* state. UBM measurements of STS have been used to predict postoperative vault and guide the selection of the lens (8, 9). However, owing to the poor repeatability of the UBM and the subjective effects of the operator on the accuracy of the measurements, it is not sufficient to use the indicators measured by this instrument alone for vault prediction. Therefore, other predictors need to be explored. To optimize the choice of lens in the clinic, the post-ICL vault needs to be predicted by combining the measurements of multiple devices and considering the complexity of the patient’s ocular structure. CASIA2 anterior segment optical coherence tomography (OCT) is the next-generation of frequency sweep OCT, which can automatically and objectively measure anterior chamber width (ACW), angle-to-angle (ATA), crystalline lens rise (CLR), and a series of other parameters without contact (10). NK and KS prediction formulas in the device can also automatically analyze the postoperative predicted vault and recommend the implanted ICL size to facilitate clinicians’ selection. These formulas have better predictability than the STAAR formula (11, 12). However, annually, 0.2–0.49% of patients undergo secondary surgery for lens removal or replacement owing to inappropriate lens size (13). In order to make the prediction formula more reliable and accurate, improve surgical safety, and reduce the incidence of secondary surgery, this study derived a new vault prediction formula for ICL lens implantation based on two instrumental measurement parameters and validated it to improve the ideal range of vault after ICL surgery and optimize the selection of lens size.

## 2. Methods

### 2.1. Population

The part involving the derivation of the formula was a retrospective study, whereas that involving its validation was a prospective cohort study. All subjects underwent ICL (EVO ICL Model V4c; STAAR Surgical) implantation at the Optometry Center of Hunan Provincial People’s Hospital in 2021–2022. For the formula derivation, the patients were assigned to the training group, and for the formula validation, the patients were assigned to the validation group. Both eyes were selected for double eye surgery, and the operated eye was the subject eye for monocular surgery. The study was approved by the Ethics Committee of Hunan Provincial People’s Hospital (Grant No. 2022-141) and complied with the Declaration of Helsinki. All patients signed an informed consent form. The study was registered with the Chinese Clinical Trial Registry (ChiCTR2200065501).

Inclusion criteria for the training group: age 18–45 years, stable diopter for >2 years (diopter <0.50 D per year), horizontal placement of all non-astigmatic lenses, horizontal placement of the lens or horizontal rotation axis of the astigmatic lens ≤10°, clear and accurate measurement of the UBM graphs with the software, and vault range of 250–1,000 μm at 3 months after surgery. Inclusion criteria for the validation group: Age 18–45 years, stable diopter for >2 years (diopter <0.50 D per year), clear and accurate measurement of the UBM-obtained graphs with the software, and timely completion of follow-up visits. Exclusion criteria: ACD <2.7 mm, corneal endothelial cell count <2,000/mm^2^, cysts of the ciliary body, previous history of refractive surgery, keratoconus, corneal endothelial dystrophy, and any other ocular disease with a corneal pathology that affects visual acuity and instrumental examination. Patients’ visual acuity, intraocular pressure, and computerized optometry were routinely measured, and postoperative vault and the presence of other ocular complications were subjectively examined using a slit lamp. Postoperative vault was objectively assessed using CASIA2 anterior segment OCT.

### 2.2. Measurement method

#### 2.2.1. Eye examination

**Figure 1:**
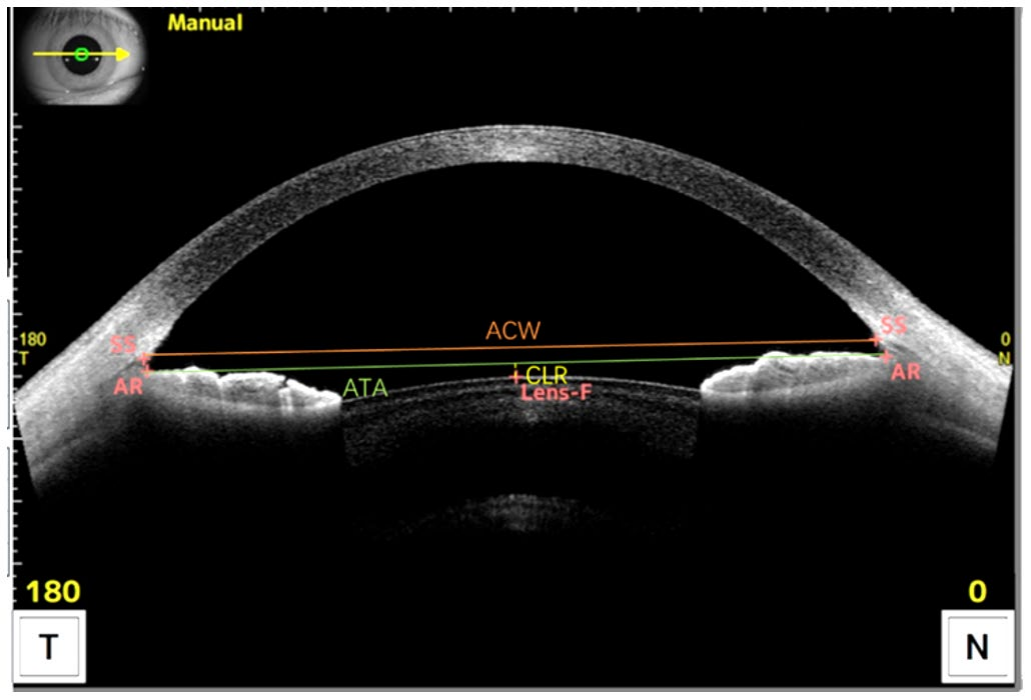
Measurement of the anterior segment parameters with CASIA2 OCT. ACW was defined as the distance between the scleral spurs on the nasal and temporal sides. ATA was defined as the distance between the angle recesses on the nasal and temporal sides. CLR was defined as the anteroposterior distance between the anterior crystalline lens surface and the angle recess to angle recess line. SS, Scleral Spur; AR, Angle Recess; Lens-F, anterior surface of the crystalline lens. The solid orange line is ACW, the solid green line is ATA, and the dashed yellow line is CLR.

The complete history of all patients was obtained, and they all underwent preoperative eye examination, including subjective and objective optometry in cycloplegic and mydriatic states, intraocular pressure measurement with a noncontact tonometer, corneal endothelial cell density measurement, slit lamp microscopy, and routine fundus examination. The horizontal-visible iris diameter (HVID) and the photopic pupil diameter (PPD) were measured using the Sirius 3D Comprehensive Eye Ganglion Analyzer (CSO, Italy). A biometric instrument, AL-Scan (NIDEK, Japan), was used to measure the axial length (AL) and horizontal WTW diameter of the eye. ATA, ACW, ACD, and CLR were measured with CASIA2 OCT of the anterior segment (Tomey, Japan) (Figure 1). The vault was measured using the CASIA2 anterior segment OCT and was automatically derived from the instrument measurement. To avoid the influence of light and accommodation on the vault, all measurements were recorded in a natural light environment with a stable light source (14). The measurements were performed by an experienced physician, and the average of three measurements was used for all markers.

#### 2.2.2. UBM image acquisition process

**Figure 2:**
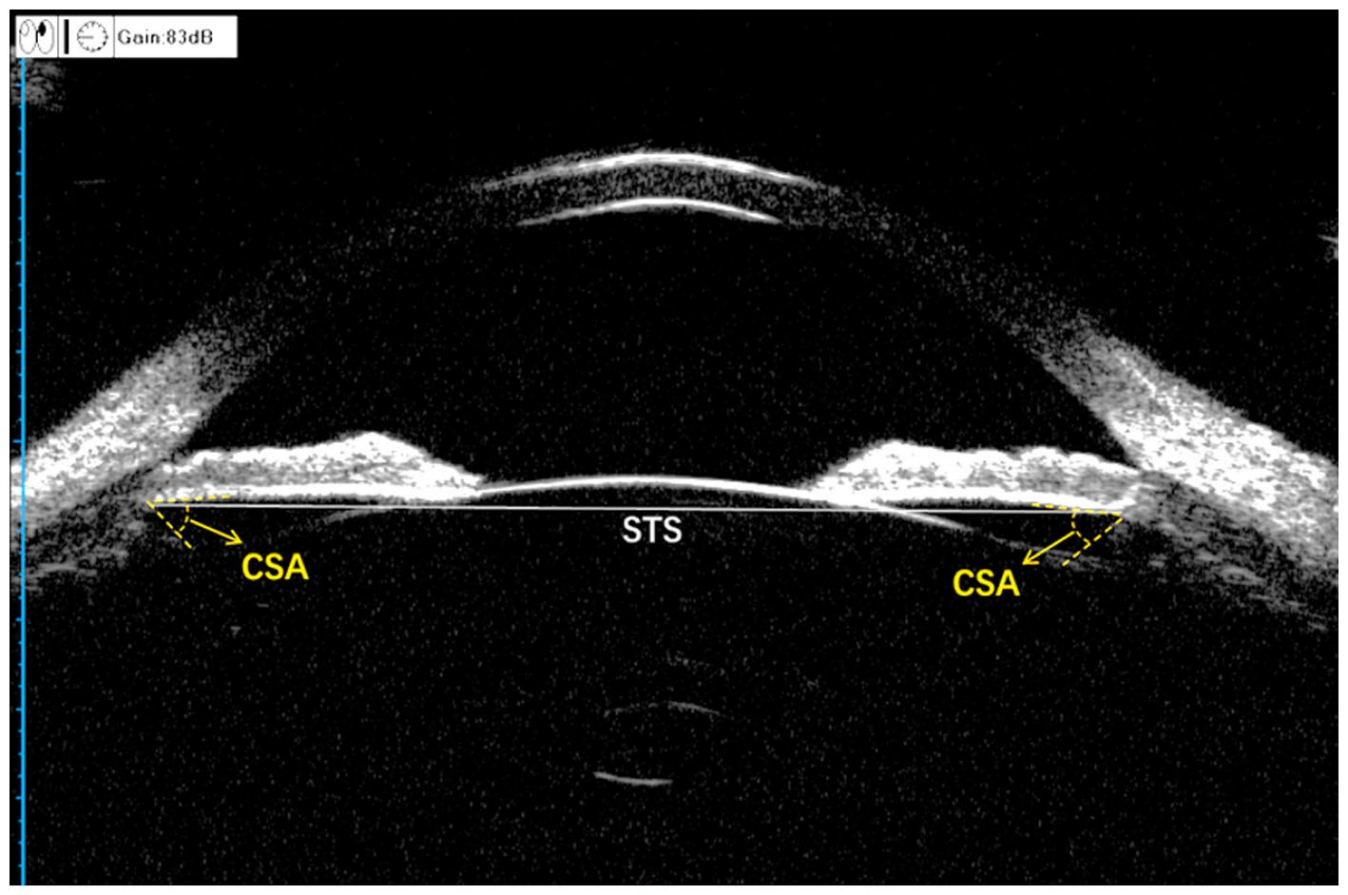
Measurement of the anterior segment parameters with UBM. CSA, ciliary sulcus angle; STS, sulcus-to-sulcus. The gray solid line is STS and the angle formed by the two yellow dashed lines is CSA.

All UBM images were acquired and the horizontal STS distance (HSTS) was measured by a physician with 8 years of testing experience. A panoramic ultrasound biomicroscope (Tianjin, SUOER SW-3200) equipped with a 50 MHz probe was used to measure the patient. The patient was instructed to lie in the supine position on the examination bed. After surface anesthesia was administered, a suitable eye cup was selected and placed in the conjunctival sac and an appropriate volume of saline was poured in it to begin the examination. With the probe placed perpendicular to the corneal apex and the non-examined eye gazing at the ceiling above, the eye position was centered and panoramic images of the anterior segment of the eye were obtained at 3–9 and 6–12 o’clock positions. The patient’s examined eye was tilted to the side, with the probe perpendicular to the corneoscleral rim, and scanned images were obtained at four positions: 2, 4, 8, and 10 o’clock. The scan was repeated several times until the image clearly showed the anterior and posterior surfaces of the cornea and the lens as well as the ciliary sulcus; fully-exposed images were included. HSTS was measured using the built-in software. All UBM images were imported to a personal computer, and the ciliary sulcus angle (CSA), defined as the angle formed by the posterior surface of the iris and the anterior surface of the ciliary body, was measured manually by another physician (Hao Wu) using ImageJ software while masking the patient data. We analyze CSA images when they meet the following criteria: (1) The ciliary sulcus is within the line of focus so that it is completely exposed. (2) The iris is tangential to the anterior surface of the lens. (3) The following anatomical signs must be clearly visible: the corneal reflective line, the lens suspensory ligament reflective line, and the ciliary epithelial reflective line (Supplementary Figure S1). Both HSTS and CSA were manually measured three times and averaged at the end (Figure 2).

### 2.3. Surgery method

All ICL implantations were performed by the same surgeon (Hua Wang). Patients were administered 0.3% gatifloxacin drops continuously for 3 days before the surgery. Eyes treated with ToricV4c ICL (TICL) were marked for astigmatism axially next to the cornea before the surgery. They were then dilated with compound tropicamide drops 40 min before the surgery. The conjunctival sac was fully flushed before the surgery, and the main incisions were all selected to be 3.0 mm temporal clear corneal incisions. The ICL was placed in the manufacturer’s dedicated pusher and implanted into the anterior chamber with the pusher. After the ICL was fully expanded, sodium hyaluronate was injected into the anterior chamber and the four loops of the ICL were adjusted to the posterior ciliary sulcus of the iris with the lens alignment hook. Sodium hyaluronate in the anterior chamber was replaced by flushing with sodium lactate Ringer’s solution, and tobramycin dexamethasone drops were administered to the operated eye immediately after the surgery. The following eye drops were administered after the surgery: tobramycin dexamethasone four times a day for 7 days; pranoprofen four times a day for 2 weeks; and artificial tears four times a day for 2 weeks.

### 2.4. Formula derivation, verification, and grouping

#### 2.4.1. Training group

For formula derivation, the NK and KS formulas that come with the CASIA2 anterior segment OCT were used as references to select the lens size. The NK formula is based on ACW and CLR (15), whereas the KS formula is based on ATA (12). The size was recommended automatically by the system’s built-in software at the end of the CASIA2 anterior segment OCT measurement of the patient. The achieved vault at 3 months after the surgery in the training group was used as the dependent variable, and the predictors, namely, ICL size, lens refraction (ICL power), HVID, PPD, WTW, AL, ATA, ACW, ACD, CLR, HSTS, and CSA, were used as independent variables. Univariate linear regression analysis was first used to evaluate the relationship between each parameter of the eye and the vault. The WH (Wang-Hua) prediction formula was then derived using multiple linear regression analysis (backward regression); the coefficient of determination (*R*^2^) was used to assess the model fit, the Durbin-Watson test was used to assess whether each sample was independent and the variance inflation factor was used to assess the presence of multicollinearity among the model independent variables.

#### 2.4.2. Validation group

The patient’s lens size was selected with reference to the prediction formula, and different lens sizes (12.1 mm, 12.6 mm, 13.2 mm, and 13.7 mm) and the patient’s ocular parameters were substituted in the WH formula to obtain different predicted vaults with a predetermined target vault of 500 μm, i.e., the lens size with the predicted vault closest to 500 μm was the recommended size. The achieved vault was recorded 1 month after the surgery, and the orientation of lens implantation was the same as that of the training group.

### 2.5. Statistical methods

All data were analyzed and processed using the software SPSS version 25.00. The normal distribution of the data was tested using the Shapiro–Wilk test. Independent samples *t*-test was applied to compare normally distributed continuous variables. Mann–Whitney *U*-test was used to analyze skewed continuous variables. Chi-square test was used to compare categorical variables. Multiple linear regression analysis was used to derive the vault prediction WH equation. Paired t-test was used to evaluate the differences between achieved and predicted vaults. Percentages (%) were used to express qualitative data. The Bland–Altman plot was used to evaluate the agreement between the achieved and predicted vaults. A difference with *p* < 0.05 was considered to be statistically significant.

## 3. Results

### 3.1. Basic information of the two groups

A total of 100 patients (179 eyes) were included, of which 35 (61 eyes) were in the training group and 65 (118 eyes) were in the validation group. Preoperative baseline information of the patients and the sizes of the lenses selected for the surgery are shown in Table 1.

**Table 1:** Population demographics and preoperative ocular dimensions.

	Training group	Validation group	*p* value
Eye, *n*	61 (35)	118 (65)	
Age, y	24.48 ± 5.72 (18 to 36)	26.56 ± 5.05 (18 to 43)	0.130^b^
Gender M/F,%/N	17% (6)/83% (29)	12% (8)/88% (57)	0.352^c^
ICL power, D	−10.49 ± 3.33 (−3.5 to −19.25)	−10.27 ± 2.48 (−4.00 to −18.00)	0.640^a^
ICL/TICL, *n*	43/18	85/33	0.480^c^
ICL Size, mm			0.125^c^
12.1	19.7% (12)	22.8% (27)	
12.6	59.0% (36)	66.9% (79)	
13.2	21.3% (13)	10.3% (12)	
13.7	0%	0%	
CLR, μm	17.97 ± 228.51 (−467 to 492)	34.80 ± 139.06 (−326 to 250)	0.240^b^
ACW, mm	11.67 ± 0.28 (11.09 to 12.20)	11.72 ± 0.42 (10.38 to 13.02)	0.588^b^
ACD, mm	3.35 ± 0.27 (2.82 to 3.92)	3.23 ± 0.24 (2.74 to 3.74)	0.816^b^
ATA, mm	11.64 ± 0.27 (11.07 to 12.05)	11.67 ± 0.42 (10.50 to 12.55)	0.518^b^
CSA,°	54.34 ± 19.11 (13 to 124)	53.65 ± 16.37 (10 to 120)	0.824^b^
HSTS, mm	11.56 ± 0.28 (11.02 to 12.10)	11.53 ± 0.40 (10.40 to 12.61)	0.619^a^
PPD, mm	3.95 ± 0.58 (2.62 to 5.14)	3.89 ± 0.56 (2.56 to 5.88)	0.463^a^
HVID, mm	11.85 ± 0.39 (11.01 to 12.67)	11.79 ± 0.38 (10.69 to 12.86)	0.363^b^
WTW, mm	11.77 ± 0.34 (10.80 to 12.30)	11.79 ± 0.40 (10.7 to 12.9)	0.604^b^
AL, mm	27.10 ± 0.86 (25.13 to 29.11)	26.75 ± 1.31 (23.48 to 29.79)	0.088^a^

ICL, implantable collamer lens; D, diopters; PPD, photopic pupil diameter; HVID, horizontal visible iris diameter; WTW, horizontal white-to-white diameter; ACD, anterior chamber depth; ATA, horizontal angle-to-angle diameter; ACW, anterior chamber width; HSTS, horizontal sulcus-to-sulcus diameter; CLR, crystalline lens rise; AL, axial length; CSA, ciliary sulcus angle. ^a^Independent *t*-test. ^b^Mann-Whitney *U* test. ^c^Chi-square test.

### 3.2. Derivation of the WH prediction formula

The achieved 3-month postoperative vault in the training group was 663.94 μm ± 165.02 μm. With vault as the dependent variable and ICL size, ICL power, HSTS, CSA, ATA, ACW, ACD, CLR, HVID, pupil, WTW, and AL as independent variables, the key factors influencing vault according to univariate linear regression analysis were (Table 2): ICL size (*R*^2^ = 0.206, *p* < 0.001), CSA (*R*^2^ = 0.124, *p* = 0.015), ATA (*R*^2^ = 0.103, *p* = 0.028), ACW (*R*^2^ = 0.135, *p* = 0.011), and CLR (*R*^2^ = 0.265, *p* < 0.001). Subsequently, multiple linear regression analysis was used to finally incorporate ICL size, ATA, CLR, and CSA into the WH vault prediction formula based on the inter-instrument coefficient of determination (*R*^2^) (Table 3). The final WH equation was vault (μm) = 414.98 × ICL size (mm) −111.78 × ATA (mm) − 0.59 × CLR (μm) −3.12 × CSA (°) −3,119.43, and the adjusted coefficient of determination (adjusted *R*^2^) of this prediction formula is 0.67, *p* < 0.001. The Durbin-Watson result is 1.777, which proves that the samples are independent. The ICL size, CSA, ATA, and CLR variance inflation factors were 1.759, 1.003, 1.811, and 1.127, respectively, which indicates the absence of multicollinearity in the four parameters.

**Table 2:** Single regression analysis for determining WH formula in the training group.

Instrument	Variable	*R* ^2^	*p*
	Size	0.206	0.001
	ICL power	0.016	0.403
UBM			
	HSTS	0.071	0.071
	CSA	0.124	0.015
AS-OCT			
	ATA	0.103	0.028
	ACW	0.135	0.011
	ACD	0.305	<0.001
	CLR	0.265	<0.001
Sirius			
	HVID	0.053	0.121
	PPD	0.037	0.193
AL-Scan			
	WTW	0.020	0.342
	AL	0.000	0.910

**Table 3:** Multiple linear regression analysis for determining WH formula in the training group.

Instrument	Combination of variables	*R*^2^	*p*
AS-OCT	ATA, ACW, CLR	0.347	<0.001
UBM	CSA, HSTS	0.182	0.012
UBM + AS-OCT	HSTS, CSA, CLR, ICL Size	0.666	<0.001
UBM + AS-OCT	CSA, CLR, ACW, ICL Size	0.664	<0.001
UBM + AS-OCT	CSA, CLR, ATA, ICL Size	0.667	<0.001

UBM, Ultrasound biomicroscopic; AS-OCT, anterior segment-optical coherence tomography; ATA, horizontal angle-to-angle diameter; ACW, anterior chamber width; HSTS, horizontal sulcus-to-sulcus diameter; CLR, crystalline lens rise; CSA, ciliary sulcus angle.

### 3.3. WH formula validation

**Figure 3:**
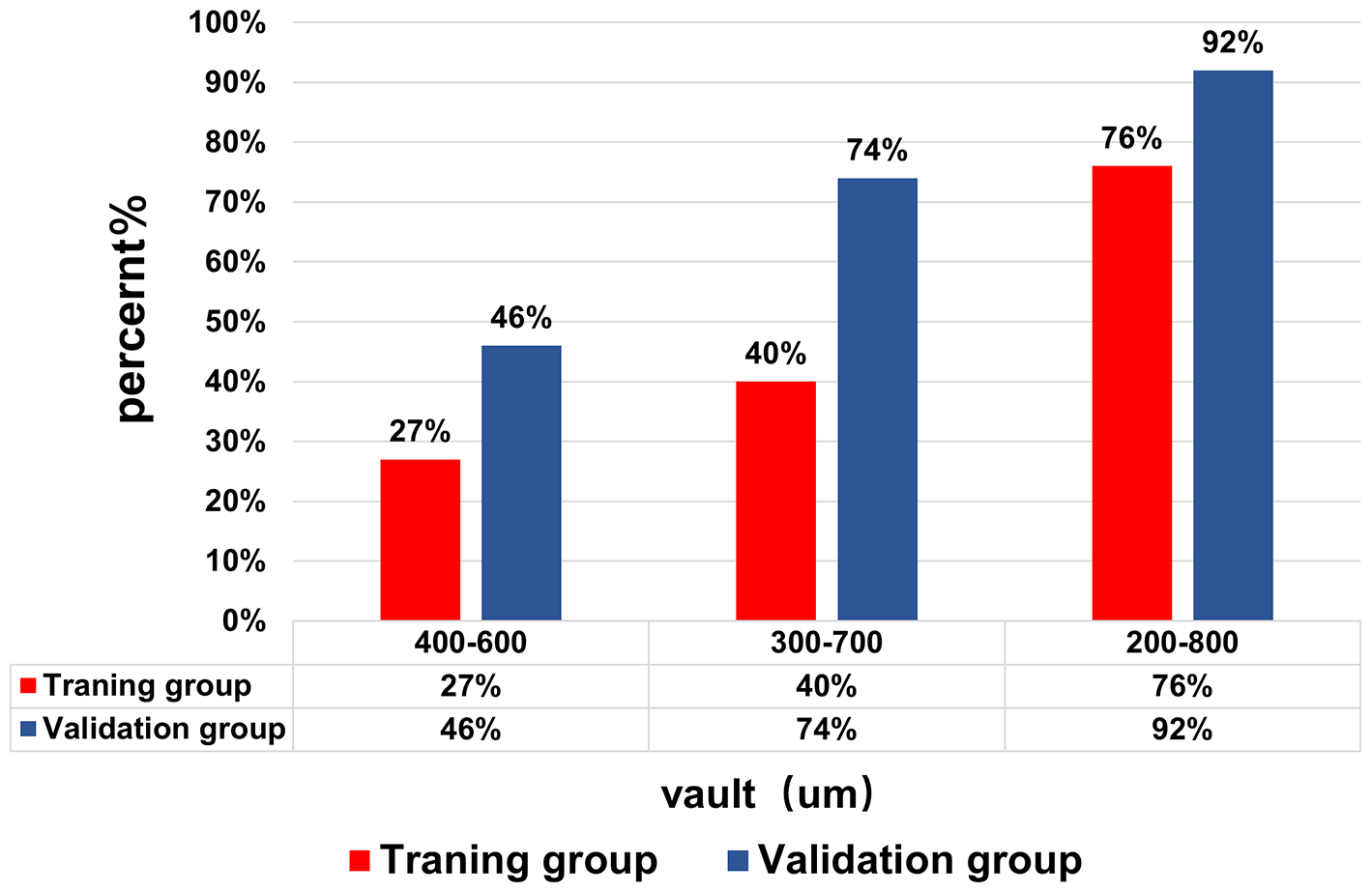
The achieved vault of the training group and validation group. The percentage of the Vault range (400–600), (300–700), and (200–800) μm in the training group and validation group.

The achieved vault 1 month after the surgery in the validation group was 556.19 μm ± 166.98 μm, range 232–1,100 μm, and 92% of patients achieved the ideal vault, and the ideal vault range was 200–800 μm. Figure 3 shows the percentages of achieved postoperative vault in the ranges of 400–600 μm, 300–700 μm, and 200–800 μm in both groups. No patient had a vault of <150 μm, but one operated eye had a vault of >1,000 μm, with normal postoperative atrial angle opening intraocular pressure and an ACD of 2.24 mm on follow-up. The patients were treated under observation. They did not develop postoperative complications related to ASC, acute angle-closure glaucoma, increased intraocular pressure, or pupillary block.

### 3.4. Comparison of the WH formula with other formulas

#### 3.4.1. Vault

**Figure 4:**
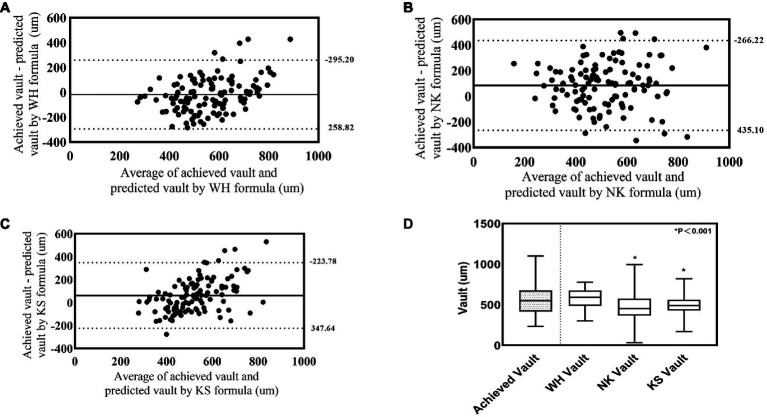
Comparison between achieved vault and model predicted Vault in the validation group. **(A)** Bland-Alterman plot of achieved vault versus WH formula predicted vault. **(B)** Bland-Alterman plot of achieved vault versus NK formula predicted vault. **(C)** Bland-Alterman plot of achieved vault versus KS formula predicted vault. The solid line represents the mean difference between achieved vault and predicted vault dotted lines are the upper and lower borders of the 95% LoA (mean difference ± 1.96 multiplied by standard deviation of the mean difference). **(D)** Box plot of achieved and predicted vault by WH formula, NK formula and KS formula.

The achieved postoperative vault in the validation group was 556.19 μm ± 166.98 μm, and the vaults predicted using the WH formula, NK formula, and KS formula were 574.38 μm ± 119.31 μm, 471.75 μm ± 152.56 μm, and 494.25 μm ± 104.63 μm, respectively. The difference between the achieved vault and that predicted using the WH formula was −18.19 μm ± 141.32 μm, and the difference was not statistically significant (*p* = 0.165). However, the differences with the NK formula and KS formula were 84.44 μm ± 178.91 μm and 61.93 μm ± 145.77 μm, respectively, which were statistically significant (*p* < 0.001, *p* < 0.001). Figures 4A–C show the Bland–Altman plots of the achieved postoperative vault in the validation group versus the vaults predicted using the WH, NK, and KS formulas, respectively. The differences between the achieved postoperative vault and those predicted using the WH, NK, and KS formulas in the validation group were −18.19 μm ± 141.32 μm, 84.44 μm ± 178.91 μm, and 61.93 μm ± 145.77 μm, respectively. In the validation group, the Bland–Altman plot showed that the 95% limits of agreement between the achieved vault and the vault predicted using the WH formula was narrower (−295.20–258.82 μm) compared with the ranges of the vaults predicted using the NK and KS formulas, and the mean line of the difference was close to the zero line. Figure 4D shows the box plots of the achieved postoperative vaults in the validation group versus the vaults predicted using the WH, NK, and KS formulas. The output vault values from the four prediction equations were derived from the dimensions selected for the actual surgery.

#### 3.4.2. Size

**Figure 5:**
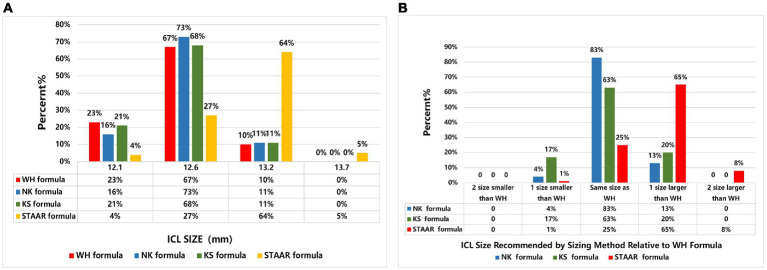
Comparison of recommended size for WH, NK, KS, and STAAR formula. **(A)** Frequency histogram of Implantable Collamer Lens (STAAR Surgical) size used based on WH formula versus size recommended by each of the principal published sizing methods (NK, KS, and STAAR). **(B)** Comparison of Implantable Collamer Lens (STAAR Surgical) size recommended by principal published sizing methods relative to the WH formula.

Figure 5A shows the size recommended by the WH formula, i.e., the percentage of the actual size selected in the operation and the sizes recommended by NK, KS, and STAAR formulas. Figure 5B shows the recommended sizes of the NK, KS, and STAAR formulas that were one or two sizes larger and one or two sizes smaller than those recommended by the WH formula and the percentage of the same sizes selected. In terms of the recommended size, NK, KS, and WH formulas had the highest percentages of the recommended size of 12.6 mm (73, 68, and 67%, respectively). The STAAR formula selected the highest percentage of the size 13.2 mm (64%). In terms of size recommendation differences, the selection size of the WH formula was closest to the NK formula recommendation size, with 83% of the same size being selected. The WH formula and STAAR formula had the greatest difference and the two formulas only have 25% selection rate for the same dimensions. The STAAR formula tended to select one size larger than the WH formula and this accounted for 65%.

## 4. Discussion

In this study, we first retrospectively analyzed patients who underwent ICL implantation and found that the main preoperative parameters that affected the postoperative vault were ICL size, CSA, CLR, and ATA. We applied CSA to the vault prediction formula for the first time. The formula was validated and compared with other prediction formulas to achieve good predictability and safety.

The WH formula derived in this study has a good safety profile and improves the ideal range of the postoperative vault. Rancons (6) and Zhang (16) used WTW and ACD to select the lens size based on the STAAR formula, and 53 and 65%, respectively, of the subjects achieved postoperative vault in the ideal range. Moshirfar (17) used STS and lens rise to select the lens size, and 77% patients achieved the ideal vault. Malyugin (18) used PTP (iris pigment end to iris pigment end distance) for size selection, and 55% of the patients achieved the ideal vault after surgery. However, in our study, 92% of the validation group achieved an ideal vault range after surgery, which is better than previous results.

The coefficient of determination *R*^2^ of the prediction formula reached 0.67 in this study, which was greater than the prediction formula of other studies. As most of the traditional vault prediction methods are based on a single instrument, the index is relatively singular and does not take into account the eye morphology, which leads to low predictability. Lee et al. (19) predicted the vault based on OCT of the anterior segment of the eye, which yielded a regression formula *R*^2^ of only 0.144. Zheng et al. (20) derived the vault prediction formula based on corneal topography and UBM, with a corrected *R*^2^ of 0.35, which was lower than our prediction formula. Although the results of univariate regression analysis in this study showed a maximum *R*^2^ of 0.305 for ACD, we did not include ACD as a predictor. This exclusion is mainly because we believe that ACD does not directly affect the postoperative vault of ICL, but it does affect the tolerable range of the vault. If the anterior chamber of the patient is shallow, ICL lens implantation will cause it to become shallower, and the chamber angle will be narrower than that before the surgery. Then, the tolerable postoperative vault range would be limited and may be in the range of 250–600 μm. A height of >600 μm may be associated with the risk of anterior chamber angle closure (21). Smaller the preoperative ACD, narrower the range of the vault that can be tolerated, and the corresponding predicted vault should be lower. Therefore, it is recommended that a combination of predicted vault values and the patient’s ACD be considered for ICL sizing.

The ICL is implanted in the ciliary sulcus, and size selection should theoretically depend more on the size of the STS. However, in this study, the univariate regression analysis of HSTS and postoperative vault was not statistically significant (*p* = 0.071); hence, we excluded HSTS from the regression model. The results of the study of Lee (4) and Reinstein (11) agree with ours, with no statistically significant relationship between postoperative vault and HSTS. A meta-analysis by Packer concluded that there was no significant difference in the use of STS and WTW to predict vault size (3). We believe that the main reason for the deviation between theoretical and actual clinical results is the poor reproducibility of the UBM instrument and the fact that the accuracy of the measurement is susceptible to the subjective effects of the operator, which increases the error of the STS measurement. In addition, there is no uniform clinical standard for the determination of anatomical sites for STS. If STS results measured by other central methods are used for vault prediction, it will produce large errors that are detrimental to the promotion of the formula. To replace STS, we incorporated ATA into the prediction equation. Several studies (12, 22) have concluded that ATA is more correlated with HSTS than WTW. The measurement of the predictive formula ATA was performed in this study using the CASIA2 anterior segment OCT, which has been shown to exhibit good reproducibility in measuring ocular segment parameters (23). For ATA measurement, CASIA2 is more accurate than other anterior segment analyzers because it avoids subjective errors associated with HSTS measurements (24).

Furthermore, ciliary sulcus morphology is important for ICL lens size selection. Zhou et al. (25) concluded that wider the ciliary sulcus, smaller the postoperative vault, narrower the ciliary sulcus morphology, and larger the postoperative vault. Patients with anterior ciliary body and ciliary process hypertrophy are at an increased risk of high postoperative vault, and patients with a wide ciliary sulcus are more likely to undergo rotation after TICL lens implantation (26). Therefore, the morphology of the ciliary sulcus needs to be considered during lens selection. In this study, the UBM examination images were reanalyzed to obtain a quantitative index of CSA, which was included in the ICL vault prediction formula for the first time. We believe that CSA is a good quantitative assessment of the ciliary sulcus morphology. The mean value of CSA in the normal population was noted to be 66.3° in Sugiura’s study (27). In the study by Chen et al. (28), the CSA was found to be 48.23° ± 16.15° in the population with normal vault and 26.18° ± 16.32° in that with abnormal vault (>1,000 μm). Moreover, the vault showed a negative correlation with CSA, which is in line with our findings. In other words, larger the CSA, wider the ciliary sulcus pattern and smaller the postoperative vault. Also, smaller the CSA, narrower the ciliary sulcus pattern and higher the postoperative vault. After ICL lens implantation, the ideal foot plate position is that it should be sufficiently located in the ciliary sulcus. Zhang’s study (16) found that the position of the lens foot plate after ICL surgery may fall above or below the ciliary sulcus, thus affecting the vault. In three patients with excessive vault, the postoperative foot plate was positioned above the ciliary sulcus. We believe that the size of the CSA affects the position of the postoperative ICL foot plate. When the CSA is small, it prevents adequate contact between the foot plate and the ciliary sulcus, which causes the foot plate to lie above the ciliary sulcus and increase the vault. When the CSA is large, it reduces the support of the ciliary sulcus to the ICL lens and may cause the foot plate to move downward postoperatively, which results in a reduction in vault (Supplementary Figures S2, S3).

We also included the CLR measured using OCT of the CASIA2 anterior segment in the prediction formula, which is defined as the vertical distance between the line connecting the apex of the horizontal cornea–iris angle and the apex of the anterior surface of its own lens. Higher the CLR, lower the postoperative vault. NK (15) used ACW and CLR for lens size selection, and 91.2% of the patients predicted by this formula achieved ideal vault after surgery. STAAR’s recommended formula only uses the WTW and ACD markers to select the lens size. CLR and ACD are negatively correlated (29), i.e., a high CLR may result in a small ACD, and if the STAAR formula is followed, a small lens size may be selected. In practice, it is found that the vault will be low at this time, which will increase the risk of ASC after surgery.

We compared the WH formula with other formulas. With regard to size, 19% of the actual size selected by the validation group was exactly the same as that recommended by the three formulas (NK, KS, and STAAR). The actual size selected was close to the size recommended by the NK and KS formulas, and the ratios of selecting the same size were 83 and 63%, respectively, whereas the rate of choosing the same size compared with the STAAR formula ratio was only 25% (Figure 5B). The prediction formula proposed by Reinstein (11) had a 40% identity rate compared with the STAAR formula. The reason is that the STAAR formula was proposed in the early days of using the nonporous V4 model to prevent anterior subcapsular cataracts due to low postoperative vault. However, the new porous V4C model, with its small central 360 μm hole, promotes posterior atrial aqueous circulation and greatly reduces the incidence of anterior subcapsular cataracts (3). Therefore, we suggest that STAAR should adjust its own size selection formula. In terms of vault, the results of Bland–Altman plot analysis showed that in the validation group, the 95% limits of agreement between the achieved vault and that predicted using the WH formula was narrower than the range of the vault predicted using the NK and KS formulas. Furthermore, the mean curve of the difference was closer to the zero curve, with better agreement (Figure 4). This may be related to the differences in the predictors incorporated between the different formulas; the predictors of the NK and KS formulas (ACW, CLR, and ATA) are all anterior chamber parameter markers that did not include the morphology of the posterior chamber. In addition, because STAAR has not published the vault prediction formula, we cannot obtain the vault predicted using this formula.

The limitation of this study is that the sample size of the formula derivation group is relatively small, and even though the coefficient of determination is high, more subjects are needed for the optimization of the formula. At the same time, in order to ensure sufficient sample size, both eyes were included for study in some subjects instead of randomly selecting an eye from every subject. We conducted a single-center study, and differences exist in ciliary sulcus anatomy and corneal diameter between ethnic groups (30). Owing to the relatively small corneal diameter in Asians, there were relatively few options for large-sized lenses in the validation group. This study did not directly provide a formula for predicting the lens size because there are currently only four sizes available for surgery at STAAR, which are not continuous numerical variables. However, the clinician can personalize the size of the lens based on the vault value output from our prediction formula combined with different patient conditions. The ciliary sulcus of the human eye is vertically elliptical, and the different directions of lens placement also exert an important effect on the vault. The vault output values of the formula in this study were proposed on the premise that the lens is implanted horizontally. If the output of two adjacent sizes is too large or too small for the vault, the surgeon may choose to implant the larger size of the crystal obliquely or vertically.

## 5. Conclusion

This study combined the results of OCT and UBM measurements of the anterior segment of the eye and incorporated the quantitative index of ciliary sulcus morphology, CSA, in the prediction formula. This was combined with ICL size, ATA, and CLR to derive a new vault prediction formula, which verified that the ideal range of postoperative vault of patients is high. We achieved optimal design of postoperative vault of ICL, improved the safety of ICL surgery, and provided a reference for ICL clinicians.

## Data availability statement

The raw data supporting the conclusions of this article will be made available by the authors, without undue reservation.

## Ethics statement

The study was approved by the Ethics Committee of Hunan Provincial People’s Hospital (Grant No. 2022-141) and complied with the Declaration of Helsinki. All patients signed an informed consent form to participate in this study.

## Author contributions

HaW and D-jZ performed the literature review and selection. HaW, D-jZ, D-qL, and HuW designed the study. HaW drafted the manuscript. HuW and D-jZ reviewed and approved the manuscript. L-yZ and JL performed the statistical analyses. All authors have read and approved the final version of the manuscript.

## Funding

This study was supported by grants from the Department of Science and Technology of Hunan Province (2020SK2118) and the Department of Education of Hunan Province (22A0044), China.

## Conflict of interest

The authors declare that the research was conducted in the absence of any commercial or financial relationships that could be construed as a potential conflict of interest.

## Publisher’s note

All claims expressed in this article are solely those of the authors and do not necessarily represent those of their affiliated organizations, or those of the publisher, the editors and the reviewers. Any product that may be evaluated in this article, or claim that may be made by its manufacturer, is not guaranteed or endorsed by the publisher.

## Abbreviations

ICL: implantable collamer lens
D: diopters
PPD: photopic pupil diameter
HVID: horizontal-visible iris diameter
WTW: horizontal white-to-white diameter
ACD: anterior chamber depth
ATA: horizontal angle-to-angle diameter
ACW: anterior chamber width
HSTS: horizontal sulcus-to-sulcus diameter
CLR: crystalline lens rise
AL: axial length
CSA: ciliary sulcus angle
UBM: ultrasound biomicroscopy
AS-OCT: anterior segment optical coherence tomography
