# Supplementary material for: Improvement in the ideal range of vault after implantable collamer lens implantation: a new vault prediction formula
Source: Front Med (Lausanne). 2023 Apr 27;10:1132102. doi: 10.3389/fmed.2023.1132102 (PMC10174235; doi:10.3389/fmed.2023.1132102)
Supplement: Supplementary file 1 [file Data_Sheet_1.DOCX]

**Supplemental Figure 1 This figure shows a clear CSA with the direction of 10 o'clock. The following conditions are met.** (1)The ciliary sulcus is within the line of focus so that it is completely exposed. (2)The iris is tangential to the anterior surface of the lens. (3)The following anatomical signs must be clearly visible: the corneal reflective line, the lens suspensory ligament reflective line, and the ciliary epithelial reflective line.

**Supplemental Figure 2 shows a 23-year-old female patient with a large preoperative CSA. Three months after surgery, we found that the foot loop was not located in the ciliary sulcus but on the ciliary body below the ciliary sulcus, and the vault was low (120 um), and this patient was temporarily managed under observation.** The left image shows the foot loop position and vault, and the right image shows a partial magnification of the foot loop position, with the white arrow showing the foot loop position, which can be found on the ciliary body below the ciliary sulcus.

**Supplemental Figure 3 shows a 33-year-old female patient with a small preoperative CSA. One month postoperatively we found that this patient's foot loop was located above the ciliary sulcus and the foot loop was tightly attached to the posterior surface of the iris, resulting in a high postoperative vault (1330 um), and we performed a size change in this patient.** The left image shows the footloop position and arch height, and the right image shows a partial magnification of the footloop position, with the white arrow showing the footloop position, which is found to be above the ciliary sulcus and the ICL lens is tightly attached to the posterior surface of the iris.
