# Supplementary figures and images for: Improvement in the ideal range of vault after implantable collamer lens implantation: a new vault prediction formula
Source: Front Med (Lausanne). 2023 Apr 27;10:1132102. doi: 10.3389/fmed.2023.1132102 (PMC10174235; doi:10.3389/fmed.2023.1132102)

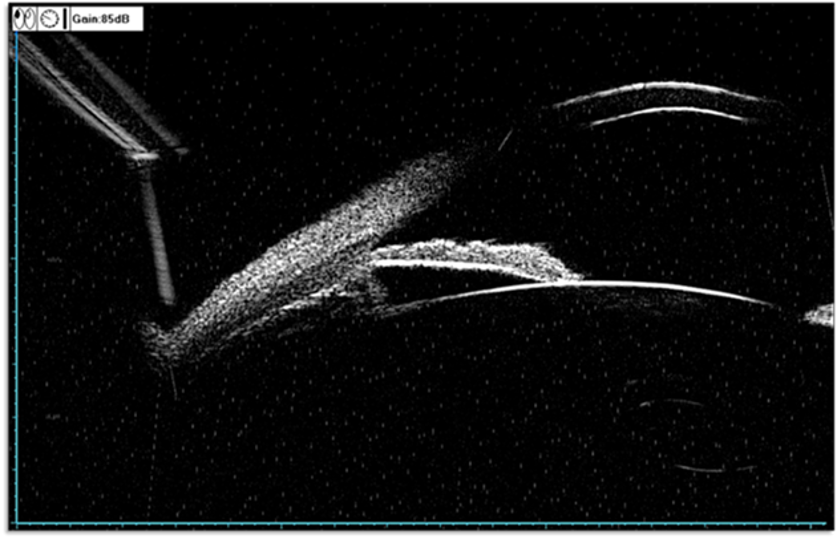

Supplement: Supplementary file 2 [file Image_1.TIF]

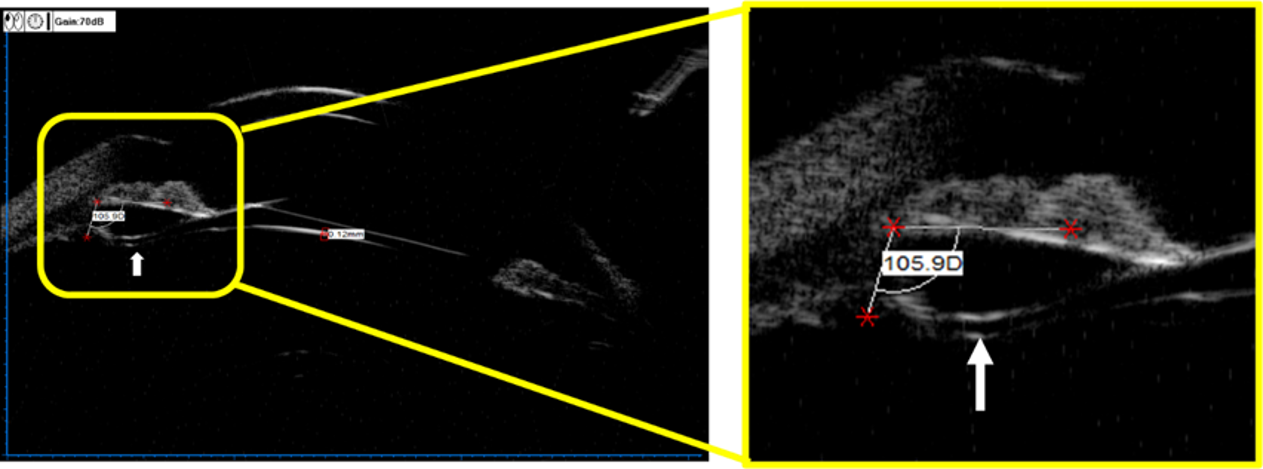

Supplement: Supplementary file 3 [file Image_2.TIF]

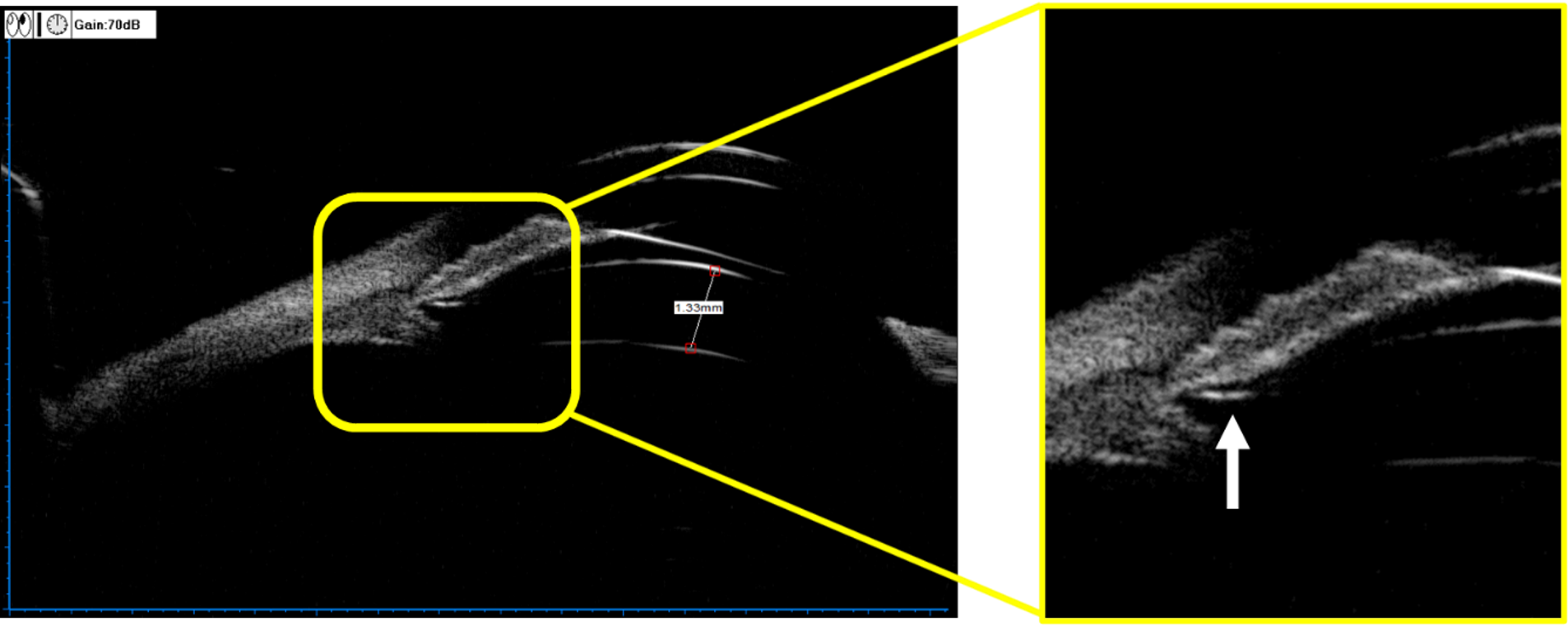

Supplement: Supplementary file 4 [file Image_3.TIF]
